# Supplementary material for: Asthma in a prospective cohort of rural pregnant women from Sri Lanka: Need for better care during the pre-conceptional and antenatal period
Source: PLoS One. 2022 Jul 14;17(7):e0269888. doi: 10.1371/journal.pone.0269888 (PMC9282538; doi:10.1371/journal.pone.0269888)
Supplement: S1 Table — *New onset is defined as an asthmatic patient who did not have a particular symptom during the last three months preceding the pregnancy developing the symptom. (DOCX) [file pone.0269888.s001.docx]

**Table 5. Demographic characteristics of patients who reported increased frequency or new onset dyspnoea or wheeze in the first and second trimester**

| **Demographic characteristic** | **Increased frequency of dyspnoea** | | | | **Increased frequency of wheeze** | | | | **New onset dyspnoea*** | | | | **New onset wheeze*** | | | |
| --- | --- | --- | --- | --- | --- | --- | --- | --- | --- | --- | --- | --- | --- | --- | --- | --- |
|  | **First trimester** | | **Second trimester** | | **First trimester** | | **Second trimester** | | **First trimester** | | **Second trimester** | | **First trimester** | | **Second trimester** | |
|  | **n** | **%** | **n** | **%** | **n** | **%** | **n** | **%** | **n** | **%** | **n** | **%** | **n** | **%** | **n** | **%** |
| **Age category (years)** | | | | | | | | | | | | | | | | |
| <20 |  |  | 1 | 11.1 | 3 | 5.7 | 3 | 13.6 | 1 | 6.3 | 1 | 6.3 |  |  | 2 | 16.7 |
| 20-24 |  |  | 1 | 11.1 | 13 | 24.5 | 10 | 45.5 | 3 | 18.8 | 2 | 12.5 | 1 | 16.7 | 2 | 16.7 |
| 25-29 |  |  | 2 | 22.2 | 16 | 30.2 | 7 | 31.8 | 7 | 43.8 | 5 | 31.3 | 1 | 16.7 | 3 | 25.0 |
| 30-34 | 1 | 100 | 4 | 44.4 | 12 | 22.6 | 2 | 9.1 | 3 | 18.8 | 4 | 25.0 | 2 | 33.3 | 4 | 33.3 |
| 35-39 |  |  | 1 | 11.1 | 7 | 13.2 |  |  | 2 | 12.5 | 3 | 18.8 | 2 | 33.3 | 1 | 8.3 |
| 40-44 |  |  |  |  | 2 | 3.8 |  |  |  |  | 1 | 6.3 |  |  |  |  |
| **Highest education level** | | | | | | | | | | | | | | | | |
| Up to grade 10 |  |  |  |  | 8 | 15.4 | 1 | 4.5 | 1 | 6.7 | 2 | 12.5 | 1 | 16.7 |  |  |
| Grade 11 | 1 | 100 | 5 | 55.5 | 22 | 42.3 | 12 | 54.5 | 5 | 33.3 | 5 | 31.3 | 1 | 16.7 | 6 | 50.0 |
| Grade 12-13 |  |  | 2 | 22.2 | 7 | 13.5 | 2 | 9.1 | 3 | 20.0 | 3 | 18.8 | 2 | 33.3 | 1 | 8.3 |
| Post-school education |  |  | 2 | 22.2 | 15 | 28.8 | 7 | 31.8 | 6 | 40.0 | 6 | 37.5 | 2 | 33.3 | 5 | 41.7 |
| **Highest education level of partner** | | | | | | | | | | | | | | | | |
| Up to grade 10 |  |  | 1 | 11.1 | 10 | 18.9 | 4 | 18.2 | 3 | 18.8 | 2 | 14.3 | 1 | 16.7 | 2 | 16.7 |
| Grade 11 | 1 | 100 | 5 | 55.6 | 30 | 56.6 | 11 | 50.0 | 7 | 43.8 | 5 | 35.7 | 2 | 33.3 | 5 | 41.7 |
| Grade 12-13 |  |  | 3 | 33.3 | 13 | 24.5 | 7 | 31.8 | 6 | 37.5 | 7 | 50.0 | 3 | 50.0 | 5 | 41.7 |
| **Gravidity** | | | | | | | | | | | | | | | | |
| 1 |  |  | 2 | 22.2 | 21 | 39.6 | 5 | 22.7 | 6 | 37.5 | 5 | 31.3 | 2 | 33.3 | 2 | 16.7 |
| 2 | 1 | 100 | 4 | 44.4 | 13 | 24.5 | 6 | 27.3 | 4 | 25.0 | 7 | 43.8 |  |  | 7 | 58.3 |
| 3 |  |  | 3 | 33.3 | 13 | 24.5 | 11 | 50.0 | 5 | 31.3 | 2 | 12.5 | 3 | 50.0 | 1 | 8.3 |
| ≥ 4 |  |  |  |  | 6 | 11.3 |  |  | 1 | 6.3 | 2 | 12.6 | 1 | 16.7 | 2 | 16.7 |
| **Parity** | | | | | | | | | | | | | | | | |
| 0 |  |  |  |  | 2 | 6.3 | 1 | 5.9 |  |  | 2 | 18.2 |  |  |  |  |
| 1 | 1 | 100 | 5 | 71.4 | 15 | 46.9 | 8 | 47.1 | 7 | 70.0 | 6 | 54.5 | 1 | 25.0 | 8 | 80.0 |
| 2 |  |  | 2 | 28.6 | 13 | 40.6 | 8 | 47.1 | 3 | 30.0 | 2 | 18.2 | 3 | 75.0 | 2 | 20.0 |
| 3 |  |  |  |  | 1 | 3.1 |  |  |  |  |  |  |  |  |  |  |
| 4 |  |  |  |  | 1 | 3.1 |  |  |  |  | 1 | 9.1 |  |  |  |  |

*New onset is defined as an asthmatic patient who did not have a particular symptom during the last three months preceding the pregnancy developing the symptom
